# Evidence of association between hepatitis C virus genotype 2b and nosocomial transmissions in hemodialysis centers from southern Brazil

**DOI:** 10.1186/1743-422X-10-167

**Published:** 2013-05-29

**Authors:** Naylê Maria Oliveira da Silva, Fabiana Nunes Germano, Raul Andres Mendoza-Sassi, Hector Nicolas Seuánez, Marcelo Alves Soares, Ana Maria Barral de Martinez

**Affiliations:** 1 Faculdade de Medicina, Universidade Federal do Rio Grande, Rio Grande, Brazil; 2 Departamento de Genética, Universidade Federal do Rio de Janeiro, Rio de Janeiro, Brazil; 3 Programa de Genética, Instituto Nacional de Câncer, Rio de Janeiro, Brazil

**Keywords:** Hepatitis C virus, Genotype, Hemodialysis, Nosocomial infection, Southern Brazil

## Abstract

**Background:**

Hepatitis C virus infection is a serious public health problem. Hemodialysis is considered one of the main risk factors of HCV infection, due to several invasive medical procedures and potential nosocomial transmission that patients with chronic renal failure (CRF) are continuously submitted. The aims of this study were to determine the prevalence of HCV and its genotypes in patients with CRF in hemodialysis units in southern Brazil.

**Methods:**

Demographic data and risk factors for HCV transmission were collected and analyzed. These data were obtained from patients undergoing hemodialysis treatment from January 2009 to August 2010, on two dialysis units of Rio Grande, southern Brazil. Genotyping was carried out by sequencing analysis of HCV NS5b, core-E1 junction and 5′UTR genomic regions.

**Results:**

One hundred fifty-nine patients under regular hemodialysis treatment were studied. HCV prevalence was 23.3%. HCV-infected patients had been on dialysis treatment for 91.9 months, a more prolonged period compared to HCV-negative patients (p = 0.001). While HCV genotypes 1b and 3a were identified as the most frequent strains, a surprisingly high proportion of genotype 2b was observed among patients in one of the dialysis centers compared to the general HCV-infected population of the same area. Hemodialysis treatment exposure time and healthcare working were associated with HCV infection.

**Conclusions:**

Besides the efforts to minimize nosocomial transmission of HCV, some events of transmission are still evidenced in dialysis units.

## Background

Approximately 170 million people worldwide are infected with the hepatitis C virus (HCV) and are at risk of developing chronic hepatitis, cirrhosis and hepatocellular carcinoma [1,2]. Moreover, HCV infection is the most common indication for liver transplantation in developed countries [3].

Brazil has an intermediate prevalence of hepatitis C, varying from 2.5% to 10% [4]. Recently, a nationwide and comprehensive cross-sectional, population-based survey has reported an HCV seropositivity rate of 1.38% [5]. Nevertheless, in the southern region of the country, anti-HCV positive confirmed cases are higher than the national average (9.4 *versus* 5.4 per 100,000 subjects in 2011, respectively) [6].

Hemodialysis is considered one of the major risk factors for HCV infection, due to numerous vascular access procedures, periodic blood transfusions and potential nosocomial transmission to which patients with chronic renal failure (CRF) are continuously subjected [1,7]. In spite of effective control and safe medical practices implemented to reduce the risk for transmission of infectious diseases among hemodialysis patients, sporadic outbreaks in dialysis units still occur. The major procedures to prevent HCV nosocomial transmission include protocols for handling bodily fluids, isolation policies and use of erythropoietin to minimize blood transfusions [7,8]. Some factors, such as blood transfusions and duration of hemodialysis treatment, have been particularly related to a higher mortality rate among those patients [9-13].

HCV infection varies by patient characteristics, geographic location, socioeconomic aspects, number of patients per dialyzer and rigorous use of the strictest biosafety standards [14,15]. Differences in patient behavior and community exposure factors may also contribute to the higher prevalence of HCV in hemodialysis units [15]. HCV prevalence among Brazilian hemodialysis patients ranges from 11% to 90% but HCV genotype characterization is not well documented [16-21]. HCV genotype 1a is the most prevalent, followed by 1b and 3a [21-25], except for the study by Busek and colleagues [20] who found genotype 2b as the second most prevalent.

Chronic renal patients have particular features that impair HCV diagnostics. Slight increases in aminotransferases levels, intermittent viremia and negative anti-HCV serology for a long period after infection are characteristics commonly observed in this population [7,19]. HCV RNA detection by RT-PCR is the best method to diagnose HCV infection in patients with CRF [26,27], despite the intermittent viremia described in 33% to 67% of anti-HCV positive patients [7].

The aims of this study were to determine the prevalence of HCV and its genotypes in patients with CRF in two hemodialysis centers of Rio Grande, southern Brazil, and the main risk factors associated with infection in this patient group.

## Results

The population analyzed comprised 57.2% of males, the mean age was 56.9 years (SD ± 15.9), and 65.4% were Caucasians. With respect to education, 66.7% were illiterate or had not completed elementary school. The main reported risk behaviors for HCV were blood transfusion (94.3%) and surgical procedures (90%). Approximately 2% of the patients reported hemophilia, 2% were intravenous drugs users (IDU), 3% shared syringes or needles and 2% informed use of inhaled cocaine.

HCV prevalence in the analyzed dialysis units was 23.3% (37/159). The mean age among HCV positive patients was 54.9 years (SD ± 13.3) and 62.2% were male. The statistical model for HCV infection was adjusted for age. Table 1 shows the crude and adjusted PRs for HCV infection according to HCV risk factors. As observed, the multivariate analysis revealed a significant PR for healthcare workers of 4.26 (p = 0.048).

**Table 1:** Crude (cPR) and adjusted (aPR) prevalence ratios for HCV infection according to risk factors (n = 159)

**Characteristic**	**Frequency total**	**Frequency HCV**^**+**^	**cPR (95% CI)**	***p***	**aPR (95% CI)**	***p***
**Age (years)**	-	-	0.98 (0.97 – 1.07)	0.32	0.98 (0.97 – 1.00)	0.19
**Sex**			1.23 (0.68 – 2.21)	0.5	1.19 (0.67 – 2.16)	0.53
Male	57.2	62.2				
Female	42.8	37.8				
**Education**			0.71 (0.37 – 1.39)	0.32	0.64 (0.34 – 1.21)	0.18
≤ 11 years	89.9	94.6				
≥ 12 years	10.1	5.4				
**Surgery**			0.92 (0.37- 2.27)	0.87	0.86 (0.35 – 2.11)	0.73
Yes	89.9	89.2				
No	10.1	10.8				
**Blood Transfusion**			2.16 (0.33-14.09)	0.42	1.93 (0.32- 11.56)	0.47
Yes	94.3	97.3				
No	5.7	2.7				
**Tattoo and piercing**			0.60 (0.09- 3.80)	0.59	0.47 (0.12- 1.81)	0.27
Yes	4.4	2.7				
No	95.6	97.3				
**Healthcare work**			2.71 (1.24- 5.91)	0.01	2.97 (1.31- 6.75)	0.009
Yes	3.1	8.1				
No	96.9	91.9				
**Injection and inhaled drug use**			1.44 (0.28 - 7.37)	0.66	2.69 (0.18- 38.31)	0.46
Yes	1.9	2.7				
No	98.1	97.3				
**Syringe sharing**			1.76 (0.57 - 5.37)	0.32	0.75 (0.11- 4.93)	0.76
Yes	3.1	5.4				
No	96.9	94.6				
**Time of hemodialysis (**mo.)	-	-	0.99 (0.98 - 1.00)	0.23	0.99 (0.99 - 1.00)	0.09

The average time of hemodialysis among all patients enrolled in this study was 55.1 months (SD ± 58). By contrast, HCV-positive patients had been on dialysis treatment for 92 months (SD ± 82), a time period significantly higher if compared to HCV-negative patients (44 ± 43 months; p = 0.001). In the multivariate analysis depicted in Table 1, this variable also showed borderline significance (p = 0.09) as independently associated with HCV infection. Whereas pre- and post-dialysis urea, potassium, calcium and phosphorus levels did not differ between HCV-negative and positive patients (data not shown), mean serum alanine aminotransferase (ALT) was significantly higher among infected patients (17.1 *versus* 27.6 IU/L; *p* = 0.003), an expected finding.

**Figure 1:**
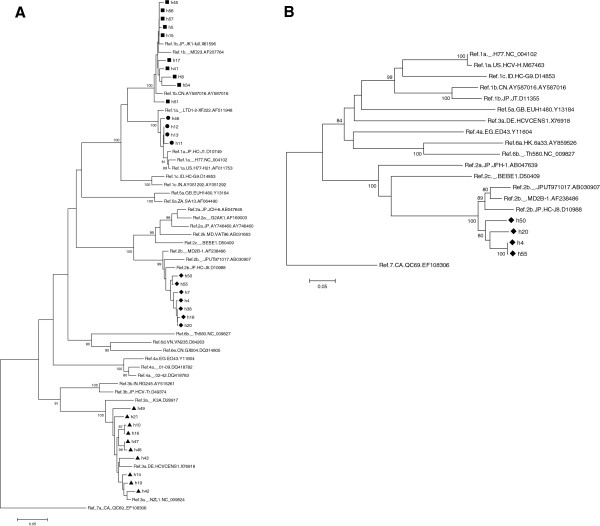
**Phylogenetic tree depicting the HCV NS5b (*A*) or the core-E1 junction (*B*) genomic region of the analyzed isolates.** HCV isolates characterized in the study are highlighted with symbols (circle, subtype 1b; square, subtype 1a; diamonds, subtype 2b; triangles, subtype 3a). Only bootstrap support values above 80% are shown, which differentiate major HCV genotypes and subtypes. The tree was rooted with a subtype 7a sequence, a variant not found in Brazil. Bars below the trees represents a genetic distance of 5% at the nucleotide level.

All serological results were confirmed by HCV RNA detection followed by PCR of NS5b or 5′UTR (Table 2). HCV genotyping was carried out in 31 (84%) of the infected patients, based on phylogenetic analysis of NS5b sequences. In six cases, HCV subtyping was not possible due to negative PCR results for this viral genomic region. These comprised four genotype 1 strains, one genotype 2 and one genotype 3. Overall, 33 5′UTR and 31 NS5b sequences were determined. For isolates for which both NS5b and 5′UTR sequences were available, both regions revealed the same genotype in all cases. According to the analysis of NS5b region, 14 belonged to genotype 1; four were subtype 1a (13%) and 10 were subtype 1b (32.5%). All genotype 2 isolates (n = 7) belonged to subtype 2b, with a prevalence of 22%. HCV genotype 3 (subtype 3a), was also present in 10 subjects (32.5%). All genotypic classifications were further confirmed by HCV 5′UTR sequencing (data not shown). A single additional genotype 2b isolated was found in Unit 1, for which only the 5′UTR genomic region was available. Figure 1A depicts all HCV NS5b fragments phylogenetically analyzed. It is evident from the tree that all genotype 2b isolates characterized in this work (all from Unit 2) clustered together, although only a fairly significant bootstrap value was achieved (70%). This cluster is not observed for the remaining genotypes found (Figure 1A), for which the sequences are interleaved with database reference sequences. However, some subtype 1a and 1b sequences are strongly similar in the analyses, and a common source of transmission in those cases cannot be completely ruled out.

**Table 2:** HCV subtype distribution in patients under hemodialysis according to phyogenetic analysis of the 5′UTR and NS5b genomic regions

**Region/Subtype**	**N**		
	**Total (%)**	**Unit 1**	**Unit 2**
5′UTR*			
**1a**	17 (52)	5	12
**1b**			
**2b**	6 (18)	1	5
**3a**	10 (30)	1	9
**NS5b**			
**1a**	4 (13)	0	4
**1b**	10 (32.5)	3	7
**2b**	7 (22)	0	7
**3a**	10 (32.5)	0	10

* For genotype 1, 5′UTR does not distinguish subtypes, and numbers are combined.

In order to corroborate the existence of a transmission cluster harboring the HCV subtype 2b sequences in Unit 2, phylogenetic analysis of another HCV genomic region (the core-E1 boundary) was carried out. A semi-nested PCR of that region was attempted for five out of the seven HCV-2b viruses observed in Unit 1, and of those four E1 sequences were generated. As shown in Figure 1B, the four sequences still clustered together with a bootstrap value of 80%, further highlighting the transmission cluster seen with NS5b sequences.

## Discussion

This is the first study characterizing the prevalence of HCV genotypes among patients undergoing hemodialysis treatment in southern Brazil. A higher prevalence of genotype 2b has been found, a genotype with rare occurrence in Brazil, what suggests a possible nosocomial transmission event in one of the two hemodialysis units studied herein. Nosocomial transmission is still a common event in dialysis units, and the proper detection of HCV infection among these patients remains a challenge.

The HCV infection prevalence found in this study (23.3%) was higher compared to previous studies in general population at the same region, which reports prevalence of 6% to 8.6% [28]. On the other hand, when studies conducted with patients on hemodialysis are considered, HCV prevalence of 14.6% to 46.7% have been reported in different Brazilian cities [16,17,19,20]. This discrepancy largely highlights HCV as a major morbidity in hemodialysis units, jeopardizing the safety of patients with CRF.

In this study, hemodialysis treatment time and healthcare occupation were significantly associated with HCV infection, the majority of them occurring in older adults and men. Meyers and colleagues [29] have also found association of HCV infection with time of hemodialysis treatment.

Several geographic and ethnic differences in the prevalence of HCV genotypes, as well as temporal, behavioral and cultural differences, have been reported [30,31]. While in Brazil HCV genotypes 1 and 3 are predominant, in some Latin American countries, like Argentina and Venezuela, genotypes 1 and 2 prevail, responding for approximately 90% of the cases [32]. In Europe, a continuous decrease of genotype 3 is observed, and an increase of genotype 2 has been registered [33,34]. The HCV genotype distribution in this study was similar to those reported by other authors for the same geographic region, with HCV genotypes 1 and 3 being responsible for most infections [35-37]. Genotype 3a has been reported as particularly common in southern Brazil [36,38]. Noteworthy, however, is the high prevalence of genotype 2b among the patients treated in the hemodialysis units enrolled here, especially from one of the units studied. This is a unique characteristic, considering the low prevalence of this genotype in Brazil [15,21,33,39,40]. These findings suggest a potential role of nosocomial transmission among the patients involved.

Hepatitis C is the most common cause of chronic viral liver disease in haemodialysis patients [17]. Several risk factors for HCV acquisition have been identified among these patients, including surgery and frequent blood transfusions they are submitted to. Furthermore, nosocomial transmission in the dialysis units may be associated with hemodialysis procedures, such as aerosol formation during fistula cannulation for vein access, accidents with contaminated blood and direct contact with contaminated materials used by infected patients [41].

Our hypothesis of nosocomial transmission of HCV genotype 2 is also supported by the phylogenetic analysis of the characterized isolates. All subtype 2b sequences clustered together for two distinct HCV genomic regions analyzed, suggesting a common source of infection. This feature was not seen for the other genotypes studied, where database reference sequences intermingled with query sequences, except for a few potential transmission links within the subtype clades. On the other hand, it is likely that subtype 2b circulates in Brazil as a monophyletic lineage, based on recent nationwide HCV sequence analyses (Lampe et al., Antivir Ther *in press*). Therefore, we cannot exclude the possibility that the subtype 2b cluster seen here reflects such single introduction of this subtype in the country, rather than a *bonafide* intra-unit dissemination. However, epidemiological data regarding the frequency of this subtype in Brazil largely argues in favor of the nosocomial transmission hypothesis. Yet this is the likely scenario in the analyzed setting, one cannot completely rule out the additional possibility that the genetic background of this variant facilitates its parenteral transmission. Paraboni and colleagues [26] found an association of HCV genotype 2b with dental procedures in southern Brazil. Added to this factor, other aspects associated to CRF patients may also be considered to explain the higher prevalence of genotype 2b, and its facilitated transmission among medical or dental interventions. Finally, yet genotype 2b is not a common variant in Brazil, it is a very prevalent genotype in neighboring countries of its southern boundaries, like Argentina, with which Brazil has an intense population interflow. Such scenario may also contribute to the higher prevalence of genotype 2b in the area.

The Brazilian Public Health System has established guidelines to prevent the spread of HCV in dialysis units [42]. These actions included rigorous implementation of universal precautions and biosafety procedures, screening for anti-HCV among patients, sterilization of dialysis machines and all instruments used and training of healthcare workers. Another important measure implemented is the isolation of patients with positive serology for anti-HCV, even during their transport or transfer, because of the risk of vascular bleeding due to heparinization during dialysis [2,15,43]. Under this scenario, it is important to know the serological status of all patients in the unit, so that measures can be implemented successfully. The difficulties on diagnosing HCV infection on CRF patients constitute the major limitation against the efforts to avoid nosocomial transmission on dialysis units. Due to late seroconversion on these patients, serological testing alone is inconclusive for HCV screening, generating false negative results [19,26,44,45].

## Conclusions

In conclusion, it has been extensively discussed that patients on hemodialysis treatment are at high risk for HCV infection. Besides the efforts to minimize or eliminate nosocomial transmission of HCV, some events can still be detected in different dialysis units. It is important to consider HCV RNA measurement in these patients, because the immunosuppression inherent to chronic renal disease, as a preventive factor against nosocomial spread of HCV.

## Methods

### Patients and study design

This was a prospective study, developed among patients undergoing hemodialysis treatment from January 2009 to August 2010, followed at two dialysis units located in the city of Rio Grande, southern Brazil. One hundred and fifty nine patients were enrolled in this survey, 116 from Unit 1 and 43 from Unit 2. The study was conducted with the approval of the local Ethics Committee of the Federal University of Rio Grande (number 7222741/2008). Written informed consent was obtained from all patients, and eligibility criteria included age over 17 years and agreement to answer a standardized questionnaire containing sociodemographic variables as well as information on risk behaviors and practices relevant to HCV acquisition.

### Samples

HCV serology, alanine aminotransferase levels and other biochemical variables were carried out at the dialysis units. Both units performed HCV screening on their patients periodically, on a quarterly basis, by a third-generation anti-HCV ELISA (Imuno-Elisa Anti-HCV, Wamma Diagnóstica, São Paulo, Brazil). During serological screening, one additional sample of 5 mL of whole blood was collected into an ethylenediamine tetraacetic acid tube from each patient enrolled in the study. Plasma was separated and sent to the Laboratory of Molecular Biology of Universidade Federal do Rio Grande (FURG) and stored at −80°C. HCV-positive samples (n = 37) were thawed and analyzed for HCV RNA detection and genotyping.

### Determination of HCV RNA and HCV genotype

Viral RNA was extracted from 140 μL of plasma with the QIAamp Viral RNA extraction kit (Qiagen, Chatsworth, USA). A final volume of 60 μL of viral RNA was mixed with 600 ng of random primers (Life Technologies, Carlsbad, USA) in diethylpyrocarbonate-treated water and incubated for 10 min at 70°C. Reverse transcription was carried out with 200 U of Moloney Murine Leukemia Virus reverse transcriptase (Life Technologies, Carlsbad, U.S.A.), 0.1 mol/L DTT, 25 U of RNaseOUT (Life Technologies) and 0.5 mmol/L of each deoxynucleotide for 90 minutes at 37°C. A semi-nested polymerase chain reaction (PCR) assay was conducted with the primers Pr3 (5′-TATGAYACCCGCTGYTTTGACTC-3′; outer foward), Pr4 (5′-GCNGARTAYCTVGTCATAGCCTC-3′; inner foward) and Pr5 (5′-GCTAGTCATAGCCTCCGT-3′; reverse) to amplify a 380 bp product corresponding to an internal region of HCV NS5b gene [46]. Additional nested PCR assays were performed with primers PTC1 (5′CGTTAGTATGAGTGTCGTGC3′) and NCR2 (5′ATACTCGAGGTGCACGGTCTACGAGACCT3′) in the first round, and with PTC3 (5′GTGTCGTGCAGCCTCCAGG3′) and NCR4 (5′CACTCTCGAGCACCC TATCAGGCAGT3′) in the second round, to amplify a 220 bp product corresponding to a region of HCV 5′UTR (Campiotto et al., 2005). Finally, for a subset of HCV subtype 2b (see Results), another semi-nested PCR reaction was carried out for amplifying a 470 bp viral genomic fragment spanning the end of the core and the beginning of the E1 genes, using the primers E1-Fow (5′GGCGTCGGTGTYAGRGTYCTGG3′), E1-1stRev (5′GGCRGTCCGRTTTATGTGCC3′) and E1-2ndRev (5′ATGACYTTGGCCCACGCTCC3′). PCR products were analyzed by electrophoresis through a 1.5% agarose gel stained with ethidium bromide.

PCR products were sequenced using the Big Dye v.3.1 kit in an ABI 3130XL Genetic Analyzer (both from Life Technologies). Sequences were manually edited with SeqMan v7.0 (DNASTAR Inc., Madison, USA) and aligned with BioEdit v.7.02316 along with HCV reference sequences from the Los Alamos National Laboratory HCV Database (http://hcv.lanl.gov). Genotypes were determined through phylogenetic inference using neighbor-joining and Kimura’s two-parameter, with 1000 bootstrap replicates, in MEGA 5.

All NS5b DNA sequences generated in this study have been deposited in GenBank and were assigned the numbers [GenBank: JQ711195-JQ711198, KC520829-KC520855]. 5′UTR sequences have not been deposited because they are shorter than 200 bp, a *sine qua non* condition for submission to the GenBank database.

### Statistical analyses

All analyses were performed with STATA® v. 8.0 (Stata Corporation, 2009). The association of time on dialysis in months and HCV infection was assessed by a Student’s *t* test. HCV prevalence and 95% confidence intervals (CIs) were estimated. Bivariate analysis was performed for calculating the prevalence ratios and CIs of several factors and outcomes. In order to adjust for potential confounders, a multivariate analysis was performed using Poisson regression and prevalence ratios (PRs) were determined.

## Competing interests

The authors declare that they have no competing interests.

## Authors’ contributions

NMOS and FNG conducted all experimental procedures. RAMS performed the statistical analyses. HNS provided reagents and infrastructure for the experimental procedures. MAS and AMBM conceived and financed the study. NMOS, FNG and MAS wrote the manuscript. All authors have read and approved the final version of the manuscript.
